# Organoids Provide an Important Window on Inflammation in Cancer

**DOI:** 10.3390/cancers10050151

**Published:** 2018-05-21

**Authors:** Kristi Baker

**Affiliations:** Department of Oncology, University of Alberta, Edmonton, AB T6G 1Z2, Canada; kbaker2@ualberta.ca

**Keywords:** cancer, organoids, inflammation, 3D structure, coculture, anti-cancer immunity, patient samples, drug screen, prognostic test, immunotherapy

## Abstract

Inflammation is a primary driver of cancer initiation and progression. However, the complex and dynamic nature of an inflammatory response make this a very difficult process to study. Organoids are a new model system where complex multicellular structures of primary cells can be grown in a 3D matrix to recapitulate the biology of the parent tissue. This experimental model offers several distinct advantages over alternatives including the ability to be genetically engineered, implanted in vivo and reliably derived from a wide variety of normal and cancerous tissue from patients. Furthermore, long-term organoid cultures reproduce many features of their source tissue, including genetic and epigenetic alterations and drug sensitivity. Perhaps most significantly, cancer organoids can be cocultured in a variety of different systems with a patients’ own immune cells, uniquely permitting the study of autologous cancer-immune cell interactions. Experiments with such systems promise to shed light on the mechanisms governing inflammation-associated cancer while also providing prognostic information on an individual patient’s responsiveness to immunotherapeutic anti-cancer drugs. Thanks to their ability to capture important features of the complex relationship between a cancer and its microenvironment, organoids are poised to become an essential tool for unraveling the mechanisms by which inflammation promotes cancer.

## 1. Introduction

The complex structure of solid cancers is a significant impediment to understanding their biology. Tumors emerge from the interplay of nascent neoplastic cells with the many other cell types in the local tumor microenvironment (TME). These include fibroblasts, endothelial and immune cells that both drive tumor growth and react to it. Inflammation fundamentally changes the nature of these interactions by reprogramming cells in ways that significantly impact cancer growth and progression [1]. Inflammation has many pro-tumorigenic effects in the body, including epigenetic reprogramming, damaging DNA, promoting hypoxia and angiogenesis, activating cancer-associated fibroblasts (CAFs), recruiting regulatory immune cells and inhibiting anti-tumor immune surveillance [1]. Many excellent reviews, including those featured in this issue of *Cancers*, provide a comprehensive look at our current understanding of the connection between inflammation and cancer [1,2,3,4,5]. However, while this link is robust, the numerous mechanisms by which inflammation can drive tumor growth remain poorly understood. This is partially due to the limited models historically available to study cell-cell interactions in a physiologically relevant context. Standard two dimensional cultures do not replicate a 3D tumor environment and cancer cell lines adapted to grow under such conditions have radically different phenotypes and functions than primary tumor cells [6]. Furthermore, the lack of 3D structure in standard tissue culture precludes the type of naturalistic coculture experiments with fibroblasts, endothelial or immune cells that are essential to understanding how these cells cooperatively regulate cancer growth. In contrast, in vivo tumor models possess the required structural context but lack the flexibility and accessibility needed for detailed mechanistic study.

The emerging field of in vitro 3D tumor models promises to circumvent many of these limitations. Although several model systems exist, they all share the key feature of embedding the cells in a matrix material that allows proliferating tumor cells to expand in three dimensions. Among these systems, “organoid” models have recently taken the center stage. Organoids are three dimensional structures of different organ-specific cell types derived from the culture of primary stem cells under conditions that lead to self-organization and spatially-restricted lineage commitment [7]. These “mini organs” thus share many structural features of the organ of interest including cell-cell junctions, cell polarity, adhesion-dependent cell communication and interactions with a complex extracellular matrix (ECM) [7,8]. Biologically, cells grown under these conditions much more closely resemble those from the tissue of interest than do cells grown in other formats and their derivation from primary tissues renders them a much more physiologically relevant model than traditional cell culture. Critically, organoids offer an unprecedented chance to study complex interactions between diverse cell types in a setting that emulates normal organ structure while being accessible to mechanistic study. Most notably, organoids can be readily generated from patient samples which can then be genetically engineered or cocultured with autologous inflammatory cells ex vivo, thereby generating a uniquely powerful human system to study how inflammation drives cancer initiation and progression. With the increasing use of immunotherapies, organoids furthermore allow both for better pre-clinical screening of novel therapeutics and for screening of patient samples to identify those most likely to benefit from these treatments.

## 2. The Organoid Technique

The biology of epithelial cells is critically determined by their structural organization and it has long been appreciated that 2D culture systems are suboptimal for studying epithelial function. Several 3D culture model systems have been developed to circumvent this problem, each with its appropriate use. Spheroids are clusters of cells grown in suspension that can be formed from either cell lines (homotypic spheroids) or dissociated tumor tissues (heterotypic spheroids) [9,10]. These structures typically lack organization and consist of undifferentiated cells, leading them to be used primarily for studying stem cells. In contrast, organoids are organized cell clusters growing in a solid matrix and intended to replicate normal cellular and structural features of the parent tissues, thereby providing an opportunity to study cell biology in a more physiologically relevant context. While organoids cannot replicate many features of a complex tissue environment, the relative ease with which they can be created and experimentally manipulated renders them a powerful model system with wide-ranging uses.

Creating organoids is a relatively straightforward process and can now be done for many different tissue types, including the normal colon, small intestine, stomach, liver, mammary glands and brain in addition to cancers derived from the colon, stomach, esophagus, pancreas, breast and prostate [6,9]. The process first involves isolating the tissue of interest and gently dissociating it to release a progenitor population of either tissue-specific adult stem cells or embryonic pluripotent stem cells. Careful tissue dissociation protocols are needed for each organ in order to maximize the ratio of stem cells to differentiated cells that can be isolated, but stringent isolation of purified stem cells is typically not required [11]. Alternatively, induced pluripotent stem cells can be used. The cells are then embedded in a 3D matrix of Matrigel, which contains laminin, entactin, proteoglycans, and collagen IV [9]. Cells are fed either with an enriched media if they derive from mesodermal tissues (e.g., intestine, liver) or minimal media if they derive from neuroectodermal tissues (e.g., brain, retina). In both cases, this media must be supplemented with tissue-specific niche factors that promote controlled differentiation and tissue organization [9,12]. For intestinal tissues, this includes factors such as epidermal growth factor, the morphogenic proteins Noggin and WNT3 and the WNT signal amplifier R-spondin [6]. For neural tissues, pluripotent stem cells must first be guided to undergo neuronal induction and can then be guided towards either forming organoids of specific brain regions using patterning factors or forming multi-regional heterogeneous organoids using self-organization factors [13]. That organoids generated in this manner faithfully replicate important features of the parent tissues is indicated by the fact that the organoids retain epigenetic marks of specific regions of the parent tissues and replicate the specific types of intercellular adhesion junctions seen for the corresponding cell types in the parent tissues [14,15,16,17]. The success of this process in establishing organoid cultures from many normal and neoplastic tissues of mouse and human origin renders it a very powerful tool for studying the factors that drive cancer development and progression.

## 3. The Benefits of Using Organoids to Study Inflammation and Cancer

Organoids offer several distinct advantages over other model systems for studying how inflammation contributes to cancer. Inflammation is a highly complex process involving multiple cells types that traffic to a particular site and engage in direct and indirect interactions with local cells that are often polarized epithelial cells [18,19]. Monolayer cultures are typically disorganized with little polarity and do not offer the chance for inflammatory cells to engage in physiologically relevant interactions or to migrate either towards or through the epithelial or tumor cells. They are, however amenable to experimental manipulations such as genetic engineering or the addition of exogenous growth factors. In contrast, in vivo models offer the best chance of examining physiological cell-cell interactions but are severely limited in how they can be manipulated. Subcutaneous tumors made from cell lines that can be relatively easily engineered do not replicate normal tissue architecture or vascular drainage patterns and thus do not offer the chance to study key processes involved in immune infiltration of tumors [20,21]. Doing so requires the use of much more challenging orthotopic tumor models, which do not exist for many types of cancer and are far less amenable to experimental modifications. Another important limitation of mouse models is that the human and murine immune systems harbor some important differences that might reduce how well observations made in animal models can be translated to human patients. In order to study human cancers in vivo, patient-derived xenografts (PDXs), in which human cancers are implanted subcutaneously into severely immunocompromised mice, are often used [22]. While host immunodeficiency is needed in this model to prevent eradication of the implanted tissues by the murine immune system, it precludes studying inflammatory processes in the tumor since critical cell types are lacking. Organoid systems make it possible to study species-specific interactions between inflammatory cells and 3D structures of primary mouse or human cancer cells. Since organoids can be genetically engineered, this flexible system enables observation of how specific cancer-associated mutations change the nature of the immune interaction [23]. Furthermore, primary organoids can be generated from normal tissues such as the intestine, mammary glands and brain [24]. This allows not only the inclusion of control conditions that dissect how inflammation differentially impacts cancer and surrounding tissues but also provides the chance to study how inflammatory cells and factors contribute to early tumorigenesis.

### 3.1. Organoids Can Be Cocultured with Inflammatory Cells

Inflammation is both an initiator and driver of cancer. Understanding how this occurs requires an understanding of the different inflammatory cell types that are present in the TME over time and that directly or indirectly contribute to cancer. These cell types include tumor-associated macrophages, tumor infiltrating lymphocytes, cancer-associated fibroblasts, endothelial cells, adipocytes and microbiota [1]. Each of these cell types can be differently distributed throughout a tumor in locations such as the lumen, the surrounding stroma or directly between the tumor cells. Since the organization of tumor cells determines what receptors they express and where these are distributed on the cell surface, studying how different inflammatory cells influence tumor biology requires them to be exposed to the cancer cells in a physiologically relevant compartment. The structural properties of the organoid culture system uniquely enable this.

**Figure 1:**
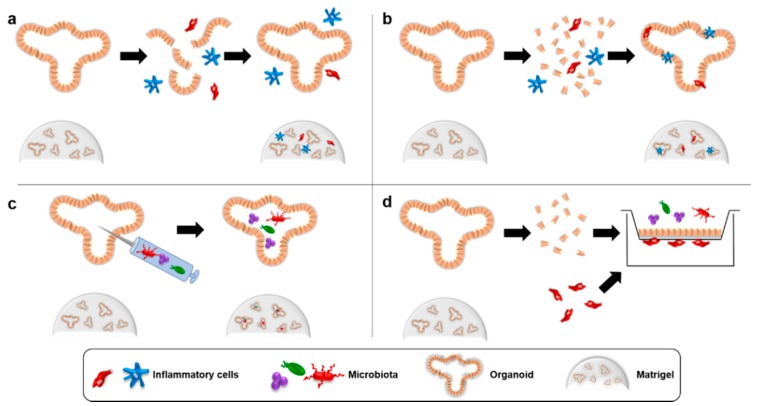
Current strategies for organoid coculture with inflammatory cells. (**a**) To study the interactions of typical stromal cells with organoids, the organoids are typically mildly dissociated and mixed with the cell population of interest in Matrigel in a method called “direct coculture”. Organoids typically rapidly reform leaving the cocultured cells in the surrounding matrix. (**b**) To study how organoid cells interact with intraepithelial cells that might invade a cancer, the dissociation of the organoids is typically more complete, often involving enzymatic digestion, and the dissociated organoid cells are mixed with the cell population of interest in media and centrifuged to bring them into immediate proximity to one another before being resuspended in Matrigel. Organoids reforming under these conditions incorporate the second cell population in direct contact with the basolateral surfaces of the organoid cells. (**c**) To study how cells normally found in a lumenal cavity interact with organoid cells, the cells of interest (typically microbiota) can be introduced into the lumen of intact organoids using microinjection techniques. (**d**) To permit more extensive manipulations, organoids can be dissociated and seeded onto Transwell inserts where the cells reform polarized layers with their apical surface facing the center of the Transwell insert and the basal surface facing the well below. For coculture with inflammatory cells, these populations can be added to the underside of the Transwell or into the upper chamber.

Coculture systems with organoids generally use one of four strategies (Figure 1). To study the interactions of typical stromal cells with organoids, the organoids are typically mildly dissociated and mixed with the cell population of interest in Matrigel, which is then plated [25,26]. Under these conditions, hereafter referred to as “direct coculture”, the organoids typically rapidly reform leaving the cocultured cells in the surrounding matrix through which they can migrate and secrete diffusible soluble factors. A variation of this method with two important changes is used to study how organoid cells interact with intraepithelial cells that might invade a cancer. The dissociation of the organoids is typically more complete, often involving enzymatic digestion, and the dissociated organoid cells are mixed with the cell population of interest in media and centrifuged to bring them into immediate proximity to one another before being resuspended in Matrigel [27]. Organoids reforming under these conditions incorporate the second cell population in direct contact with the basolateral surfaces of the organoid cells. An entirely different method must be used to study how lumenal cells interact with organoid cells. For this purpose, the cells of interest (typically microbiota) can be introduced into the lumen of intact organoids using microinjection techniques [6]. Finally, organoids can be dissociated and seeded onto Transwell inserts where the cells reform polarized layers with their apical surface facing the center of the Transwell insert and the basal surface facing the well below [28]. While less physiological, this creates a highly versatile system where different cell populations of interest can be introduced into different chambers according to their normal anatomical location. This system is particularly useful since it allows easy manipulation of conditions surrounding the cells, such as addition of trophic factors, study of cell migration across the organoid layer and evaluation of epithelial barrier integrity through measurement of the transepithelial resistance. These four strategies have allowed researchers to begin studying how cancerous or pre-cancerous cells interact with inflammatory cells in the TME.

Fibroblasts and endothelial cells in the TME are secretory cells that can play a major role in promoting tumor-associated inflammation. Several different organoid models have been adapted to study the influence of these cells on epithelial or cancer cells. For example, directly coculturing mammary gland organoids in a matrix surrounded by fibroblasts was found to induce significant branching morphogenesis [29]. Similarly, direct coculture of lung organoids with either fibroblasts or endothelial cells significantly increased the clonal proliferation and differentiation rates of the lung stem cells, with stronger effects being seen for the fibroblasts [30]. Separate experiments where genetically engineered pancreatic organoids were directly cocultured with fibroblasts further showed that WNT production by fibroblasts is essential during only the initial stages of pancreatic cancer development [25]. A similar dependence on TME cells was seen for hepatoblasts, which only proceeded to form liver buds when directly cocultured with stromal and endothelial cells because hepatic differentiation and migration only occurred with crosstalk between VEGF-secreting hepatoblasts and the endothelial cells [31]. In this experiment, direct contact was required between the blasts, stromal and endothelial cells, which initially assembled into a multi-lineage organoid before full transition to a liver bud. The contribution of fibroblasts to the gastrointestinal stem cell niche has also been explored using a variation of the Transwell assay where the fibroblasts were seeded in Matrigel directly on the permeable membrane and overlaid with a layer of Matrigel-embedded organoid cells [32]. In this setup, the fibroblasts significantly enhanced organoid growth, as did enteric neurons embedded in Matrigel directly with the organoids, indicating that both of these TME cell populations may promote cancer initiation and growth. These experiments collectively demonstrate that fibroblasts contribute to the very earliest stages of tumorigenic transformation. Additional studies have shed light on how inflammatory fibroblasts promote progression of established tumors. Experiments using both direct co-culture and Transwell co-culture between pancreatic cancer organoids and cancer-associated fibroblasts have identified two separate populations of fibroblasts within the TME that exert different tumor promoting effects [33]. One population secretes abundant proinflammatory IL-6 as a result of paracrine interactions with the cancer cells whereas the second population of fibroblasts secretes a highly desmoplastic stroma following direct juxtacrine interactions with the cancer cells. Further studies similar to these are needed to understand the mechanisms behind these observations.

Tumor-associated macrophages are dominant proinflammatory cells in the TME and their presence in a tumor is directly correlated with a poor patient prognosis [34]. While very few organoid studies have been performed on macrophages and cancers, the feasibility of such studies is evidenced by several published studies examining the interaction of macrophages with organoids formed from normal tissues. Macrophages seeded onto the underside of Transwell inserts that had an intestinal organoid monolayer on top significantly enhanced the intestinal barrier function and organoid differentiation [35]. Furthermore, when pathogenic *E. coli* were added to the apical surface of the Transwells, the macrophages promoted epithelial resistance to the pathogen and altered their inflammatory cytokine secretion pattern. In contrast, direct coculture of intestinal organoids with immune cells isolated from the lamina propria (including T cells and macrophages) led to organoid rupture in the presence of an inflammatory stimulus unless probiotic *Lactobacillus* species were added, which significantly protected the organoids from inflammation-induced injury [36]. Similar studies with tumor-associated macrophages and cancer organoids will provide vital mechanistic information regarding the contribution of this inflammatory cell population to cancer development and progression.

Additional cells contribute to inflammation-associated tumor progression and can be studied with organoid cocultures. Methods have been developed to coculture dendritic cells with intestinal organoids, which led to activation of NOTCH signaling in the organoids, and for culture of lymphocytes with intestinal organoids [27,37,38]. The latter is particularly relevant to understanding mechanisms of tumor-induced immune suppression since many of the tactics used by cancer cells to inactivate tumor infiltrating lymphocytes require direct contact via checkpoint proteins such as PD-1/PD-L1 [39]. The Transwell organoid culture system has also been used to study the interaction between adipocytes, a highly secretory cell known to promote cancer-associated inflammation, and intestinal organoids. This work demonstrated reciprocal crosstalk between the two cell populations which led them both to produce more pro-inflammatory cytokines [40]. Using organoid coculture systems, it is thus possible to study a wide range of cells that contribute to inflammation in cancer to better understand the mechanisms at play and identify promising therapeutic targets and the patients most likely to respond.

The microbiota are now recognized as an important promoter of tumorigenesis and this is largely as a result of their secretion of proinflammatory metabolites and ability to stimulate proinflammatory cells in the TME [1,41]. While the intestinal microbiota are most strongly associated with colorectal cancer, considerable evidence indicates that microbiota can similarly promote cancer at more distant body sites [41,42]. In vivo models will be needed to fully understand how the complex interactions between different microbial populations drive tumorigenesis but more reductionistic models will help dissect the molecular pathways by which individual microbes exert their effects. In this respect, organoid cultures represent an excellent system for studying how microbiota induce and promote cancer growth. To date, most microbiota-organoid cocultures have been performed in the context of infectious diseases. For example, exposure of intestinal organoids to *Salmonella* species disrupts cell-cell junctions, induces NF-κB signaling and decreases the LGR5 stem cell-associated protein [43]. *Salmonella* species also induce loss of polarity and growth factor independence in gallbladder organoids [44]. One of the few cancer-related organoid models where the influence of microbiota on carcinogenesis has been directly studied involves injection of *Helicobacter pylori* into the lumen of gastric organoids. In this work, which clearly demonstrated some of the directly transforming properties of the microbe, *H. pylori*-induced NF-κB signaling, cell proliferation and activation of the c-Met oncogene [45,46,47]. The anaerobic nature of many microbial species in the human intestine complicates studying their effect on cancer cell biology and is a significant limitation to this application of organoid cocultures. Nonetheless, at least one study demonstrated 12-h survival of normally anaerobic *Clostridium difficile* when injected into the lumen of intestinal organoids, suggesting that at least preliminary studies are possible with the current organoid technology [48]. Furthermore, exposing organoids to microbial-derived metabolites and genotoxins is currently possible and can provide valuable information about the inflammatory potential of the source microbe. Short chain fatty acids (SCFAs) such as butyrate, propionate and acetate are proinflammatory byproducts of microbial carbohydrate metabolism that induce proliferation and epigenetic alterations in intestinal organoids [6,49,50]. In addition to their proinflammatory effects, microbiota can also play an immunoregulatory role that could be studied using organoid cocultures. For example, microbial metabolites such as taurine, histamine and spermine regulate NLRP6 inflammasome activation in intestinal epithelial cells and prevent proinflammatory dysbiosis [51]. Using a gut-on-a-chip model, Kim et al. demonstrated that certain mixtures of commensal microbes were able to control pathogen growth and epithelial inflammation and similar work could be carried out and scaled up with the comparatively simple cancer organoid cocultures [52]. This type of work is particularly important in light of recent evidence that the efficacy of chemotherapy, radiotherapy and immunotherapy critically depends on the microbiota [53,54,55,56]. Since many of the pro- and anti-inflammatory effects of the microbiota occur via cross-talk with immune cells, the ability to include immune cells in the physiologically relevant basolateral compartment of cocultures between lumenal microbes and organoids renders this a particularly powerful system for mechanistically studying the contribution of microbiota to cancer-associated inflammation [36].

### 3.2. Organoids Enable Study of the Cancer-Initiating Mechanisms in Chronic Inflammatory Diseases

One of the most important and yet least studied aspects of cancer biology is the process of cancer initiation. While many of the risk factors for cancer have been documented, the molecular mechanisms leading to transformation of cells remain obscure, largely because of technical limitations such as the inability to culture completely untransformed cells. Robust organoid culture protocols now developed for normal mouse and human cells offer the opportunity to study these early events and research is currently underway for tissues such as pancreatic, bladder, kidney, colorectal and brain cancers [24,57,58].

Tumor initiation research is particularly relevant to the study of cancer-associated inflammation since many chronic inflammatory conditions predispose sufferers to cancer development [59]. The most common of these are Barrett’s esophagus, hepatitis, gastritis (from *H. pylori* infection) and inflammatory bowel disease (IBD) [11,46,60,61,62]. Organoid models exist for each of these conditions allowing for the study of inflamed pre-neoplastic patient cells as well as study of normal human tissues in the presence of suspected causative factors. The lion’s share of the work in this area has, to date, been performed for IBD. For example, studies with intestinal organoid cultures have established that Caspase-8 prevents RIP-mediated necroptosis in Paneth cells and thus may be one cause of barrier dysfunction in IBD patients [62,63]. Barrier dysfunction was also observed in organoids lacking the IBD susceptibility gene *NOD2* [64]. Furthermore, organoids from IBD patients have also been found to express high levels of IL-28R that, in turn, promotes STAT1 activation and cell proliferation [65]. Inflammation also increases the invasiveness of intestinal epithelial organoid cells deficient in TGFbR2, a gene frequently lost in a subset of colorectal cancer patients deficient in DNA mismatch repair [66]. Similarly, loss of the IBD susceptibility gene *ATG16L1* by intestinal epithelial cells promotes barrier disruption as a result of increased TNF-induced epithelial apoptosis [67]. Since loss of intestinal barrier integrity and disrupted proliferation and apoptosis are common features of colorectal cancer, these organoid-based studies collectively demonstrate several mechanisms by which proinflammatory genes associated with IBD can contribute to the early stages colorectal cancer development.

### 3.3. Organoids Can Shed Light on the Interaction between Inflammation and Cancer Stem Cells

Careful studies have clearly demonstrated that stem cells are both necessary and sufficient for organoid generation. In a landmark study, the Clevers group demonstrated that single LGR5^Hi^ stem cells from the healthy small intestine could give rise to long-term in vitro 3D cultures with a distinct crypt-villus architecture and containing all of the typical differentiated epithelial lineage cells present in vivo [68]. In contrast, LGR5^Low^ cells failed to grow, typically dying within 12 h of seeding. Similar results have been seen for organoids grown from other tissues [7]. The centrality of stem cells to organoid cultures is furthermore highlighted by the ability to generate organoids of numerous tissue types from pure iPS cell populations using appropriate growth factors [69,70,71]. Given the increasing evidence that cancer stem cells ultimately give rise to all cancers and can independently regenerate heterogenous cancers, it is important to understand how they are affected by inflammation [72].

To this effect, evidence is mounting that inflammation directly regulates the biology of cancer stem cells. This involves numerous processes and significant crosstalk between the two cell types. Cancer stem cells upregulate effectors in many key developmental signaling pathways such as Sonic Hedgehog (SHH), NOTCH and TGFβ, each of which has been associated with stem cell maintenance and activation of the epithelial mesenchymal transition (EMT) process by which cancer stem cells may arise [73,74]. EMT involves the dedifferentiation of cancer cells and is induced by upregulation of the mesenchymal transcription factors TWIST, SLUG and SNAIL in conjunction with downregulation of epithelial transcription factors FOXA1 and GATA3 [75]. Cells having undergone EMT lose their tight connections with adjacent cells by downregulating E-cadherin, develop a mesenchymal-like morphology and acquire migratory properties conducive to metastatic spread. Cells having undergone EMT phenotypically and transcriptionally resemble somatic stem cells and evidence indicates that they may serve as a continuous source of cancer stem cells for a growing tumor [72]. Inflammation within the local TME potently drives EMT.

Pro-inflammatory cytokines such as IL-6, IL-1β and TNF are produced by a variety of cell types in a cancer including the tumor cells themselves, cancer-associated fibroblasts, and tumor-associated macrophages. IL-6 in particular activates STAT3 signaling in tumor cells and high STAT3 phosphorylation is associated with a poor prognosis in many types of cancers [76]. Among the reasons for this is that the IL-6–STAT3 signaling axis in cancer cells activates NOTCH and SHH which in turn stabilize TWIST, SLUG and SNAIL by preventing their degradation [77]. Activation of these developmental pathways both promotes growth of existing cancer stem cells and induces EMT in the more differentiated tumor cell population. Similarly, the pro-inflammatory cytokine TNF induces expression of TGFβ and BMPs in the TME. TGFβ is one of the main drivers of EMT because it directly induces dedifferentiation by activating SNAIL and SLUG via stimulation of the SMAD4 pathway. TGFβ and BMPs furthermore regulate the renewal of cancer stem cells [73,78]. IL-1β, which is secreted by pro-inflammatory macrophages, activates NFκB signaling within tumor cells which, among other effects, upregulates IL-6 production thereby establishing a positive regulatory feedback loop for maintaining cells in a stem-like state. This is further perpetuated by high expression of WNT in the TME which occurs because chronic stimulation by inflammatory cytokines facilitates nuclear translocation of beta-catenin, thereby boosting cancer cell proliferation [79]. While these studies collective demonstrate the importance of inflammatory cytokines to maintaining stemness, organoids will accelerate much of the remaining work needed to identify the relevant cell populations, how they interact in the TME, what tumor-intrinsic factors regulate these processes at a molecular level and how inflammation induced stem cell growth can be therapeutically interrupted.

Mechanistically, the pro-inflammatory state of the TME induces hypoxia and acidification of the affected tissues. This in turn leads to overexpression of the HIF1a transcription factor in response to low pH, which prevents its degradation by the VHL E3 ubiquitin ligase [74,80]. HIF1a overexpression leads to upregulation of the numerous HIF1a target genes, many of which are also upregulated in cancer stem cells. Notable among these is the transcription factor TAZ, which is a downstream effector of the Hippo developmental signaling pathway driving proliferation in many stem cells. In some cell types, inflammation-induced HIF1a has been shown to directly suppress apoptosis by cooperating with NOTCH to induce the anti-apoptotic BCL-2 protein [81]. If this mechanism is also active in stem cells, it would contribute greatly to expansion of this population within a growing tumor. Inflammation is also associated with blanket changes in epigenetic regulation in cells that parallel those seen in stem cells. Several epigenetic programmers, such as Jumonji and histone acetyl transferases (HATs), are differentially regulated in inflammation. Jumonji (JMJD3), specifically, is a demethylase that has been associated with cell differentiation and is upregulated by inflammation [82]. Furthermore, there is a positive feedback loop between JMJD3 and inflammation since removal of H3K27 methylation marks upregulates specific pro-inflammatory genes. Other studies indicate that increased HAT activity at the promoters of pro-inflammatory cytokine genes IL-1, IL-2, IL-8 and IL-12 induces their expression in inflammatory diseases in an NF-κB-dependent mechanism [83,84,85]. Similar increases in the activity of HATs are seen in hematopoietic cancer stem cells [86,87]. The importance of this link between inflammation, cancer stem cells and epigenetic modification has been demonstrated in a model of colorectal cancer (CRC) [88]. Chronic intestinal inflammation was found to hypermethylate DNA regions enriched in transcription factors and developmental genes and this pattern persisted in subsequently developing adenomas and adenoma-derived organoids made from intestinal stem cells. A similar epigenetic program was identified in human CRC patients, thereby confirming the relevance of this finding in human disease. Application of organoid technology to future studies of inflammation and cancer stem cells promises to provide greater mechanistic insight into these processes and identify new feedback loops through which the process could be regulated.

## 4. Current Applications of Organoid Technology

### 4.1. Current Organoid Technology

**Figure 2:**
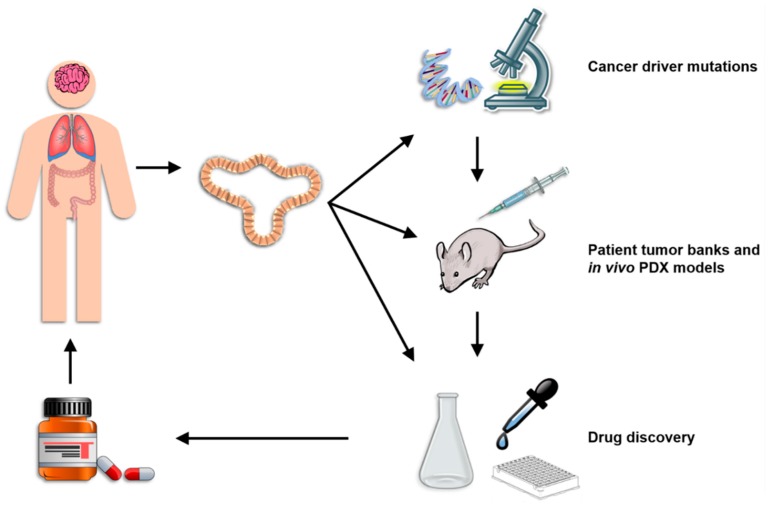
Summary of current applications of organoid technology. Patient-derived organoids can be studied molecularly to elucidate the function of driver mutations, injected into recipient mice to study in vivo growth patterns and drug sensitivity, and used in the development of novel drug compounds. The versatility of the organoid system renders them an important tool in the implementation and advancement of precision medicine.

Organoids represent a highly versatile model system that bridges the gap between in vitro and in vivo animal research models and pre-clinical and clinical human studies (Figure 2). Perhaps most importantly, organoids can be reliably genetically modified using either viral gene transfer, electroporation or liposomal transfection. Protocols have been published for each of these gene delivery methods although viral gene transduction is the most common. [89,90,91]. In all cases, the organoids are first pretreated for several days with nicotinamide to enrich for the stem cell population, dissociated into single cells during the gene introduction process, and then cultured for several days in Matrigel after gene introduction with an apoptosis inhibiting agent such as the ROCK inhibitor Y-27632. Transfection/transduction rates using any of these techniques is relatively low at 1–5% and the successfully engineered cell must be selected either by antibiotic selection or flow cytometric sorting for a fluorescent marker [89,90]. Despite these low efficiency rates, gene modification using either shRNA or CRISPR/Cas9 is widely performed using these techniques and has led to numerous interesting discoveries. For example, lentiviral delivery of shRNA against anti-inflammatory TGFbR2 in gastric cancer organoids led to metastatic differentiation and the acquisition of genetic heterogeneity, indicating that cancer-associated mutations in *Tgfbr2* drive tumor progression [92]. Furthermore, a rare and highly valuable in vivo metastasis model for colorectal cancer organoids has also been established using liposomal transfection of shRNA against APC to generate organoids that can be transplanted into recipient animals [93]. In addition, genetic engineering of pancreatic cancer organoids using electroporation to deliver sgRNA for CRISPR/Cas9-mediated deletion of multiple oncogenes has revealed that stem cell niche factors are only needed during the initial stages of tumorigenesis [25]. Combining genetic engineering of organoids with the coculture methods described above provides an extremely valuable system for studying the impact of inflammation both on the induction of driver gene mutations and on the evolution of established cancers in a proinflammatory TME.

Recent advances in organoid imaging technology will also prove invaluable to studying the dynamics of tumor cell interaction with inflammatory cells in a physiological environment. Non-invasive sectioning techniques such as multiphoton and lattice light-sheet microscopy can be applied to cocultures of organoids and immune cells to reveal structural and molecular features governing the interaction between these cell populations [94,95]. Even more powerful is the 4D imaging technology that enables dynamic monitoring of live cell-cell interactions within a living organoid coculture system [96,97]. This technique is particularly important for studying immune dynamics in patient-derived samples, where organoids can be cocultured with the immune cells isolated from the blood or tumor environment of the actual patient. Since patient-derived organoids can only be implanted into severely immunodeficient NSG (NOD-scid IL2Rgnull) mice, there is no opportunity in this system to study cancer-associated inflammation in vivo and live imaging of in vitro cocultures is the only method for obtaining this critical information.

### 4.2. Identifying the Function and Immune Consequences of Driver Mutations

Organoids are essential for the study of driver mutations in cancer since they permit the long-term culture of normal primary cells that can be genetically engineered in order to determine the earliest events in tumor initiation. For example, several research groups have used sequential introduction of driver gene mutations to identify the individual roles of each in the tumorigenic cascade. By using CRISPR/Cas9 to sequentially introduce mutations in *Apc*, *Tp53*, *Kras* and *Smad4* in intestinal epithelial organoids, Drost et al. determined that while simultaneous mutation of *Apc* and *Tp53* was sufficient to induce chromosome missegregation and aneuploidy, mutation of all four genes was necessary for full invasive growth of the tumors in vivo [90]. Similar results were later published by Li et al. who also found that pancreatic and gastric organoids exhibited sustained dysplasia with disruption of only *Tp53* and *Kras* [98]. This technique has also been used to identify new driver mutations in breast cancer organoids, identifying *Mll3* and *Ptpn22* as novel drivers in tumor progression [99]. Similarly, in pancreatic organoids, dual mutations in *Kras* and *Cdkn2a* led to non-tumorigenic clones, dual mutations in *Kras* and *Tp53* only produced tumorigenesis in the presence of cancer-associated fibroblasts and quadruple mutations in *Kras*, *Cdkn2a*, *Tp53* and *Smad4* led to rapid tumorigenesis [25]. Collectively, these studies illustrate the potential of using organoids to study how specific gene mutations alter a cancer cell’s interactions with inflammatory cells in the TME.

There is increasing recognition that the mutation profile of each cancer has specific immunological consequences for how the tumor interacts with neighboring immune cells. While this is epitomized by the well-documented relationship between mutational load and CD8^+^ tumor infiltrating lymphocyte numbers in cancers, the relationship between a tumor’s genotype and immune phenotype extends far beyond this [100,101,102,103]. Specific oncogene and tumor suppressor mutations are correlated with specific immunological parameters in the TME both within and across cancer types. For example, loss of *Tp53* is consistently associated with high macrophage and monocyte infiltration in cancers situated in tissues as divergent as the colon, lung, prostate and pancreas [101]. In contrast, *Ctnnb1* (beta-catenin), *Egfr* and *Pten* mutations are associated with decreased CD8^+^ T cell infiltration in melanoma, non-small cell lung carcinoma and prostate cancer, respectively. While identifying these links between immune infiltration and tumor genotype initially relied on laborious histological assessment of tumor sections, high throughput next generation sequencing and transcriptomics are now being used to assess the immune status of cancers [104]. This enables study of much larger sets of patient samples and publicly available datasets, thereby increasing the ability to detect potentially meaningful relationships between cancer mutations and their immune context. This will undoubtedly identify many more promising genomic alterations that might regulate anti-tumor immunity and thus affect a patient’s response rates to immunotherapies [105]. However, research in this area to date remains correlational and provides very few mechanistic insights into how oncogenic mutations regulate specific aspects of the immunological TME. Organoid coculture systems promise to shed light on the mechanisms regulating the immune effects of cancer mutations since they allow different cell populations to be cultured together for extended periods of time and can be set up from normal, untransformed cells. A related dimension that begs to be investigated in this way is to identify the mutational signatures that different inflammatory cells induce in cancers. One of the most significant ways that inflammation promotes tumor growth is by producing DNA damaging compounds such as reactive oxygen species (ROS) [3,106]. Since each genotoxic agent leaves a permanent signature pattern of mutations in exposed cells, it is possible to trace back the exposure history of a given tumor using established methodology [8,103]. Applying these techniques to long term cocultures of cancer organoids and inflammatory cells could establish mutational signatures of different inflammatory cells that could then be applied to patient samples in order to understand the immunological history of a cancer. This could provide valuable information about the likelihood of a patient to respond to immunotherapies or help identify existing immune escape strategies of the cancer.

### 4.3. Patient Tumor Banks and In Vivo PDX Models

Arguably the most important application of organoid technology is the reliable, long term ex vivo growth of human cancers in a stable system that permits multidimensional study. The lack of pre-clinical models for detailed study of the biology of human cancers has significantly limited our understanding of the disease. Living organoid biobanks of many types of cancers have now been established and many studies have documented how reliably organoids recapitulate the biology of the original tumors [24,107]. These collections of colorectal, breast, pancreatic, lung, kidney and liver cancer provide the opportunity for the first time to study in mechanistic detail patterns of heterogeneity, genetic selection, tumor growth and tumor spread in a large sample of patients [25,57,69,97,108]. Combined with the ability to genetically engineer these cells and establish coculture experiments, organoid technology is a powerful cancer research tool that is already leading to important discoveries with direct relevance to inflammation. One important feature of organoids is that they can be implanted into immunodeficient mice to permit in vivo study of the tumor. While this method can provide valuable information on many aspects of tumor biology, its use for the study of inflammatory processes is limited by the absent immune system in the host [109,110]. Several workarounds aiming to create humanized mice are currently in use to attempt to compensate for this, each with its own limitations [111,112]. In the “immune avatar model”, NSG mice are engrafted with human peripheral blood mononuclear cells (PBMCs), ideally from the same patient as the tumor, that transfer a representative sample of the donor’s peripheral immune cells to the mice. Due to interspecies immune incompatibilities, however, graft-vs-host disease invariably occurs within weeks of the engraftment. Alternatively, NSG mice can be engrafted with CD34^+^ human hematopoietic stem and progenitor cells isolated from cord blood or adult bone marrow. Recipient NSG mice become stably engrafted with adaptive and innate immune cells within several weeks and are more stable than the PBMC-reconstituted mice [109]. Besides the extremely high cost of this model, its major limitation is an immune mismatch between the tumor and immune cells since each come from a different donor, meaning that even though CD4^+^ and CD8^+^ T cells are present in the engrafted host, they cannot be effectively recognize or kill the MHC mismatched tumor cells. Furthermore, reconstituted NSG mice of both types lack the appropriate cytokine environment needed for proper immune activation since species-specific differences in these proteins do occur. While better in vivo models are currently being developed, current in vitro organoid coculture systems offer the advantage of being able to closely study the interactions between a patient’s own tumor and inflammatory cells to identify potential therapeutic targets and complications [109,113].

### 4.4. Platform for Drug Discovery

Gaining better management of patient care relies critically on the development of new drugs that more accurately target a patient’s tumor. Unfortunately, most drugs fail once they reach human clinical trials. The overall success rate of drugs entering clinical trials from 2000 through 2015 was approximately 10% [114,115]. For drugs targeting cancer, however, only a dismal 3.4% successfully passed clinical trials and made it to the bedside of patients. Better pre-clinical drug screening strategies are clearly needed. Organoids for the first time offer the chance to screen potential new drugs in living, biologically representative samples from a wide range of cancer patients. Giving our increasing understanding of the molecular variations in each cancer type and how significantly this affects patient prognosis, it is obvious that pre-clinical testing in as many primary human tumors as possible is necessary to identify patient subpopulations in which new drugs are effective, thereby increasing the overall clinical trial success rates [116].

Organoids are an excellent platform for pre-clinical drug discovery because of their versatility and the relative ease with which they can be established by research groups worldwide. In comparison to other pre-clinical models, organoids are superior in several respects. Compared to commonly used monolayer techniques with continuous cell lines, organoids are structurally and physiologically much more similar to patient tumors, maintain a stable genome over long periods of culture (including rare mutations typically lost in cell lines), have a lower therapeutic potential and more diverse response to drugs [9,117,118]. Compared to in vivo PDX models, organoids can be readily genetically engineered, are not limited by low engraftment rates that selectively expand only some subsets per tumor type, are much faster and cheaper to establish, are amenable to high throughput screening, and can be cocultured with autologous immune cells from the patient to screen immunotherapeutics [9,96,110,119]. Despite the differences in these two techniques, both organoids and PDX models show similar levels of drug response patterns when tested in high throughput screens of multiple cancer types [22]. Furthermore, both methods proved superior to simple whole tumor exome sequencing, which was found not sufficiently sensitive to identify therapeutic avenues for the majority of sample tested. A further benefit of organoids with respect to drug testing is that organoids from normal tissues can be used to measure off-target toxicity of novel drug candidates, as was recently demonstrated in nephrotoxicity tests of a group of candidate compounds using iPS-cell derived kidney organoids [120]. Numerous studies indicate that organoids successfully recapitulate patient drug response profiles. In one landmark study, not only did organoids established from sequential biopsies of a gastroesophageal cancer liver metastasis demonstrate the same patterns of primary and acquired resistance to paclitaxel as did the patient during treatment, but the organoids also recapitulated the patterns of intra- and inter-patient heterogeneity in response to TAS-102 [107]. Organoids can also be used to establish firm causative links between genetic alterations and drug responsiveness, as has been done in ovarian organoids with homologous recombination deficiencies and in liver cancer organoids with *Kras* and *Arid1a* mutations [118,121]. Similar work with patient organoids can lead to biomarker identification and validation both at the discovery level within populations and in individual patients seeking treatment. The ability to coculture organoids with different inflammatory cells thus offers the possibility of identifying specific associations between the genetics of a cancer and its response to inflammation as well as of discovering biomarkers that could inform physicians of the likelihood of a patient responding to immunotherapy or experiencing inflammatory complications. Organoids are thus a highly valuable tool in the implementation of precision medicine.

Recognizing the value of organoid technology, several groups have obtained patents for methods and technologies related to organoid use and research [122]. The first patent officially associated with the term “organoids” was granted in 2001 and pertains to a specific culturing method where a biocompatible matrix implanted into a patient becomes infiltrated by the patients own cells to form a “functional organ equivalent”. In 2014, the Clevers group who pioneered the current field of organoid research was granted a patent for a specific culturing method where epithelial stem cells from tissue fragments are grown in the presence of a bone morphogenic protein inhibitor, mitogenic growth factor and WNT agonist. The Clevers group has since established The Hubrecht Organoid Technology (HUB) organization which has a mission to “…build a comprehensive Living Biobank of well-characterized organoids and develop assays for drug screening and validation…” (http://hub4organoids.eu/) [123]. The most recent organoid related patent to be granted was in 2015 and relates to epithelial organoids containing aggregates of differentiated cell types that assume the structure and function of an epithelial organ and the use of these to treat a subject in need of a replacement of such an organ. To date, no specific patents have been filed relating to the use of organoids for cancer inflammation research or the applications thereof.

## 5. The Future of 3D Culture

Organoid technology continues to advance at a rapid pace, with appropriate culture conditions being developed for new tissues and variations of culturing techniques permitting study of more complex cell-to-cell and organ-to-organ interactions. These new systems will be critical for studying the dynamics of cancer-associated inflammation, which relies on culturing tumor and immune cells in a physiological yet experimentally malleable system. Some of the new organoid models involve relatively simple cocultures systems, such as including cancer organoids and liver organoids in separate Matrigel structures in the same well and introducing a drug that either requires metabolic activation by the liver or is metabolized by the tumor into a hepatotoxic byproduct [124]. Such a system could be highly valuable for initial toxicity screens of novel drug compounds.

Unraveling the complex interactions between immune and cancer cells requires more sophisticated methodology than coculturing but is essential for the future of cancer research and patient care. The incorporation of organoids into microfluidics systems or organs-on-a-chip offers the potential to study complex cancer-immune cell interactions and numerous approaches to this are currently being developed. In their simplest form, microfluidics devices allow for the coculture of different cell populations in defined regions that are interconnected by continuously perfused fluid-filled channels through which cells can circulate [125,126,127]. Two recent studies clearly demonstrate the enormous potential of such a system for studying cancer-associated inflammation. By embedding glioblastoma (GBM) organoids in a collagen matrix in one chamber of a device and separately introducing macrophages, Cui et al. determined that the GBM cells polarized the macrophages towards the immunosuppressive M2 phenotype by secreting TGFb [128]. Since the microfluidics device included endothelial lined vascular channels, the group also observed that both cells jointly promoted angiogenesis. Agliari et al. also used a microfluidics device to identify IRF8 as a critical driver of immune cell trafficking towards tumors [129,130]. This was done by introducing fluorescently labeled melanoma cells and splenocytes from either WT or IRF8 knockout mice into different chambers of the device that were connected by microfluidics channels and performing live cell tracking of the cells. These versatile devices can be made even more relevant to studying cancer-immune dynamics by incorporating additional cell types from the TME or metastatic sites or by using different scaffolding material that incorporates various extracellular matrix components. 

Additional technologies that can be combined with tumor organoids will enhance research into cancer-associated inflammation. Organoid culture conditions are being developed for new cell types that will broaden the range of cell-cell interactions that can be studied and allow the creation or more complex in vitro systems that come closer to modeling human physiology. Culture conditions have now been found that permit organoid formation from circulating tumor cells isolated from prostate cancer patients, raising the possibility of studying how inflammation contributes at many different stages of the metastatic cascade [131,132]. Novel 3D bioprinting methods can layer different scaffold matrices and cell types in structures approximating those seen in the human body and thereby facilitating study of immune cell trafficking into and out of the TME [71]. This will be facilitated by advances in microscopy that facilitate imaging of complex 3D structures in vitro, ideally in either high throughput format or in living cultures [94]. New developments in intravital microscopy will also enable monitoring of immune trafficking in to and out of implanted organoids, possibly in combination with lineage tracing experiments using fluorescent tags that identify both the origin and fate of inflammatory cells in the TME [133]. Such studies will identify not only the biological processes controlling cancer-immune cell dynamics but also facilitate monitoring the status of anti-tumor immunity in patients to predict response rates, toxicity or resistance to immunotherapeutic treatments.

## 6. Conclusions

Organoids are an exciting new technology that is facilitating the study of many previously inaccessible aspects of cancer biology. Included among these is the nature of cancer-associated inflammation as either an initiating or promoting factor in cancer development. Current coculture techniques between cancer organoids and inflammatory cells are already providing important insights into the dynamic relationship between these cells. The advent of new technologies will undoubtedly greatly increase the breadth of study in this field. Organoid-based research is not without important limitations such as potential artifacts induced by ex vivo culturing, the lack of a real tumor stroma and tumor vasculature, restricted or absent contact with non-tumor cell populations and the limited technology for detailed high resolution analysis of such complex 3D culture systems [6,9]. Nonetheless, organoids offer significant advantages over other systems for studying cancer and inflammation. Organoids resemble the parent tumor genetically and epigenetically even after long term culture, they respond to chemotherapeutic agents similarly to the original tumor, they can be established from normal tissues to study the initiation of cancer and they can be genetically engineered to study true mechanistic cause-effect relationships between cancer genotypes and their phenotypes. Perhaps most significantly with respect to cancer-associated inflammation, autologous organoids cocultures can easily be set up between a patient’s tumor and their immune cells in order to evaluate the status of a specific individual’s anti-tumor immune response. In the age of precision medicine, this could prove to be an invaluable tool for selecting a treatment plan that maximizes both the efficacy of immunotherapies and the endogenous immune destruction of the tumor for each patient.
